# Enhancing Galantamine Distribution in Rat Brain Using Microplasma-Assisted Nose-to-Brain Drug Delivery

**DOI:** 10.3390/ijms26041710

**Published:** 2025-02-17

**Authors:** Abubakar Hamza Sadiq, Md Jahangir Alam, Farhana Begum, Mahedi Hasan, Jaroslav Kristof, Md. Al Mamun, Md. Maniruzzaman, Kosuke Shimizu, Takanori Kanazawa, Tomoaki Kahyo, Mitsutoshi Setou, Kazuo Shimizu

**Affiliations:** 1 Graduate School of Science and Technology, Shizuoka University, Johoku, Chuo-ku, Hamamatsu 432-8561, Japan; h.s.abubakar.22@shizuoka.ac.jp (A.H.S.); mahedi.hasan.23@shizuoka.ac.jp (M.H.); 2 Graduate School of Medical Photonics, Shizuoka University, Johoku, Chuo-ku, Hamamatsu 432-8561, Japan; alam.md.jahangir.20@shizuoka.ac.jp (M.J.A.); f.begum.24@shizuoka.ac.jp (F.B.); 3 Organization for Innovation and Social Collaboration, Shizuoka University, Johoku, Chuo-ku, Hamamatsu 432-8561, Japan; kristof.jaroslav@cii.shizuoka.ac.jp; 4 Department of Cellular and Molecular Anatomy, Hamamatsu University School of Medicine, Handayama, Chuo-ku, Hamamatsu 431-3192, Japan; mamun@hama-med.ac.jp (M.A.M.); d22107@hama-med.ac.jp (M.M.); kahyo@hama-med.ac.jp (T.K.); setou@hama-med.ac.jp (M.S.); 5 Nanotheranostics Laboratory, Division of Innovative Diagnostic and Therapeutic Research, Institute of Photonics Medicine, Hamamatsu University School of Medicine, Handayama, Chuo-ku, Hamamatsu 431-3192, Japan; kshimizu@hama-med.ac.jp; 6 Graduate School of Biomedical Science, Tokushima University, Shoumachi, Tokushima 770-8505, Japan

**Keywords:** non-thermal plasma, DBD microplasma, nose-to-brain drug delivery, galantamine hydrobromide, MALDI-IMS

## Abstract

Nose-to-brain (N2B) drug delivery is a promising technique for the treatment of brain diseases. It allows a drug to enter the brain without passing through the blood–brain barrier. However, the nasal cavity and nasal mucosa can restrict the amount of drug absorbed. Recent studies of non-thermal plasma (NTP) have shown improvement in in vitro drug delivery to cells and tissues. However, whether NTP treatments can enhance the in vivo delivery of drugs for neurodegenerative disease like Alzheimer’s disease (AD) into the brain via the N2B technique remains unclear. The drug used in this study was galantamine hydrobromide. Galantamine is used to treat patients with mild to moderate AD. Based on the principle of NTP, a type of dielectric barrier discharge (DBD) plasma, which we called spiral DBD microplasma, was designed. It was inserted into the nose of a rat to a depth of 2 mm. The spiral DBD microplasma was driven by a sinusoidal voltage for 4 min, followed by the immediate administration of galantamine. The effect of the microplasma treatment on the distribution of galantamine in the brain was evaluated using matrix-assisted laser desorption/ionization-imaging mass spectrometry (MALDI-IMS). The results showed a high distribution of galantamine in the left and right brain hemispheres of the rat treated with plasma discharge compared to a control treated without plasma discharge. The spiral DBD microplasma is a novel contribution to DBD plasma designs. In addition, this technique for drug delivery has also created a novel approach with potential for becoming a non-invasive method of enhancing drug distribution in the brain for the treatment of neurological disorders.

## 1. Introduction

Neurodegenerative diseases are neuronal diseases that cause the gradual death of the nerve cells in the brain and nervous system, leading to loss of brain function over time [1]. Their causes are not fully understood [1,2], and the cures for most neurodegenerative diseases are not fully developed [3]. Aging is the primary reason for developing neurodegenerative diseases like Alzheimer’s disease (AD). AD is a common cause of dementia, leading to memory loss and the patient’s inability to think and perform daily tasks [4].

The first-line treatment for patients with mild-to-moderate Alzheimer’s symptoms is the use of acetylcholinesterase inhibitors that prevent the breakdown of acetylcholine (the brain chemical responsible for nerve cell communication) and improve cholinergic neurotransmission, which is absent in Alzheimer’s patients [5,6,7,8]. Acetylcholinesterase inhibitors include rivastigmine, donepezil, and galantamine [5]. Compared to rivastigmine and donepezil, galantamine has a dual mechanism of action. It inhibits acetylcholinesterase and modulates nicotinic acetylcholine. Since the conventional oral administration of galantamine has several limitations, like low bioavailability, rapid metabolism, high drug elimination, and gastrointestinal side effects like nausea and vomiting [4,9], there is the need for an alternative delivery route for delivering galantamine like intranasal administration.

Recent studies of intranasal administration have reported its potential as a pathway for the nose-to-brain (N2B) delivery of therapeutic agents [10,11,12]. N2B drug delivery is a non-invasive approach that enables the delivery of therapeutic agents directly into the central nervous system (CNS) [13,14,15]. However, the nasal cavity is not a uniform structure that allows the direct delivery of drugs. It contains four types of epithelia that appear in sequence in the nasal cavity. From the outermost to the innermost cavity, the nasal cavity contains the squamous epithelium, transitional epithelium, ciliated pseudostratified columnar epithelium, and respiratory epithelium [16]. The nasal epithelia affect intranasal drug delivery in several ways. This includes the slow diffusion of a drug in the mucus layer of the nasal epithelium [17] and the rapid clearing of drugs from the nasal cavity by mucociliary clearance, increasing the risk of drugs entering the gastrointestinal tract and affecting drug absorption [18]. Due to these limitations, there is need for a novel technique to enhance N2B drug delivery.

Plasma is the fourth state of matter. It has special properties compared to solids, liquids, and gasses. It is generated when gas is ionized. It is classified into thermal plasma (fully ionized) and non-thermal plasma (NTP) or cold plasma (partially ionized). NTP has received a lot of attention in recent years in the field of biology and medicine. To enable plasma to make direct contact with cells, tissue, organs, and living organisms to generate biomedical effects, a new interdisciplinary field called plasma medicine was born [19]. Plasma medicine is a multidisciplinary field that encompasses plasma physics, plasma chemistry, medicine, biology, and engineering. It focuses on the use of atmospheric plasma generated at low temperatures in biomedicine [20,21]. Previous biomedical applications of NTP include surface sterilization [22] and surface treatments to enable compatibility with biomaterials [23]. NTP has also gained recognition in novel problems such as tissue engineering [24], indoor air purification [25], water treatments [26], bacterial disinfection [27], and the sterilization of reusable heat-sensitive medical equipment [27,28]. Recent studies have also shown NTP’s direct application in medicine for the treatment of various cancer cells [29] and mammalian cells [30,31,32], cell drug delivery [33], blood coagulation [34], wound healing [35], transdermal drug delivery [36], the treatment of skin disease [37], dermatology [38], the disinfection of coronavirus [39], etc. Extensive studies have shown that the biological effect of NTP is likely due to various plasma components like free radicals (which have a lifespan of nanoseconds to seconds), charge particles (positive and negative ions), excited species, ultraviolet radiation, thermal effects, and electromagnetic fields. These generated NTP components can initiate series of biochemical reactions when in contact with living organisms, which is thus a reason for their application in the treatment of various diseases. The migration from in vitro studies to in vivo ones is still being investigated. Plasma’s discharge is strongly influenced by the nature of the biological sample with which it is in contact [40]. This is because the biological sample is part of a transient electrical circuit that is created within the high-voltage and ground electrodes during plasma discharge.

There are various methods for generating NTP. The plasma employed in this study is dielectric barrier discharge (DBD) microplasma [41]. This microplasma is a plasma of small dimensions, ranging from tens of micrometers to thousands of micrometers [41,42]. DBD microplasma is created by the electrical discharge between two electrodes separated by a dielectric barrier, which covers one of the electrodes (usually the high-voltage electrode). The gap between them results in the formation of plasma discharge. Drug delivery with DBD microplasma involves the introduction of the molecule into the target site [42]. The most recent challenge in drug delivery is brain drug delivery for the treatment of neurological diseases [43].

Due to the diverse applications of DBD microplasma for biomedical purposes, this study designed a new type of DBD microplasma to assess its suitability in enhancing the delivery of galantamine hydrobromide (HBr) into rat brains via the intranasal route (N2B). The distribution of galantamine in the rat brain was measured with matrix-assisted laser desorption/ionization-imaging mass spectrometry (MALDI-IMS). MALDI-IMS is a technique with a multiplexing capability and high sensitivity used for analyzing, identifying, and measuring biomolecules in a section of tissue [44]. It can reveal the spatial distributions of drugs and their metabolites at a microscopic scale in frozen sections [45,46,47,48]. In this study, and for the first time, we tried to enhance the N2B delivery of galantamine into rat brain with DBD microplasma. We applied two different plasma discharge conditions (1.2 kV_max_ and 1.4 kV_max_) in the left nose of a rat at room air flow rates of 0.1 L/min and 0.2 L/min. Though the plasma treatment was via the left nostril, we observed galantamine distribution with MALDI-IMS in both the left and right hemispheres of the brain.

## 2. Results

### 2.1. Plasma Discharge Characteristics

**Figure 1:**
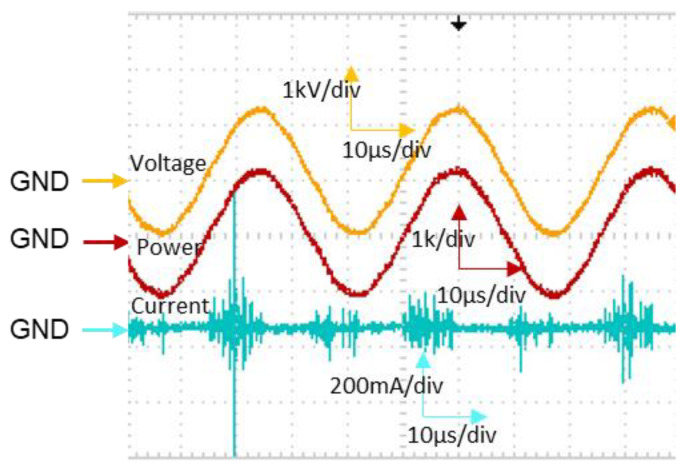
Typical waveform of the discharge voltage, discharge power, and discharge current. The GND (ground) indicates the starting point of the waveform.

The discharge voltage and current were monitored with an oscilloscope. The waveform is shown in Figure 1. The discharge voltages were set at 1.2 kV_max_ and 1.4 kV_max_ and at a frequency of 27 kHz, while the discharge currents measured were approximately 112 mA and 146 mA. The current spikes observed in the positive and negative cycles were because of streamer propagations and a glow-like discharge [49,50]. The power dissipated by the spiral DBD microplasma electrode was estimated to be 1.6 W and 3.2 W by multiplying the frequency over the integral of the discharge voltage and discharge current with respect to time. The discharge power increased with the increase in voltage. The surface temperature of the plasma electrode was measured at different times with a thermal camera. It ranged between 30 and 38 °C. The surface temperature was observed to increase when the voltage increased. This observation was due to the increase in the area where the streamers propagated. The temperature of the plasma electrode is a part of the plasma-induced effect used for enhancing drug delivery. Although the temperature could indirectly have an effect on N2B drug delivery, this was not covered in this study.

### 2.2. Concentration of Reactive Oxygen and Nitrogen Species (RONS)

NTP created in ambient air contains a high amount of reactive oxygen species (ROS) and reactive nitrogen species (RNS). Examples of ROS are ozone (O_3_), superoxide (O_2_), and hydroxyl radicals (OH^−^) while RNS are nitric oxide (NO) and nitrogen dioxide (NO_2_). Similarly to ROS, RNS also play crucial physiological functions when kept within a tolerable range. Most conditions related to impaired wound healing and microcirculation are restricted by NO bioavailability [51]. As such, exogenous NO treatment with spiral DBD microplasma could represent a novel approach in these cases. NO is an inorganic gas that reacts with oxygen to produce stable oxidative products like nitrite (NO_2_^−^) and nitrate (NO_3_^−^) [52].

**Figure 2:**
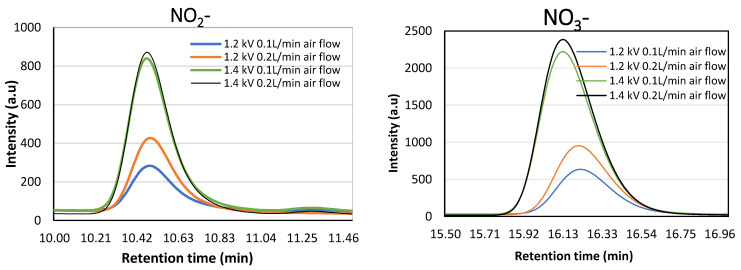
Ion chromatogram detection of NO_2_^−^ and NO_3_^−^ in distilled water treated with spiral DBD microplasma for 3 min at discharge voltages of 1.2 kV and 1.4 kV and air flow rates of 0.1 L/min and 0.2 L/min.

The NO_2_^−^ and NO_3_^−^ generated when the spiral DBD microplasma was energized were measured by ion chromatography (Figure 2). The plasma was energized in distilled water for 4 min. We observed an increase in the intensity peak as the discharge voltage increased from 1.2 kV to 1.4 kV (Figure 2). The intensity peak was also observed to increase with the increase in the air flow rate from 0.1 L/min to 0.2 L/min. The concentration of NO_2_^−^ and NO_3_^−^ calculated from the standard solution of sodium nitrite (NaNO_2_) and potassium nitrate (KNO_3_) was estimated to be −13 µM, 10 µM, 70 µM, and 77 µM, for NO_2_^−^ and 13 µM, 30 µM, 95 µM, and 103 µM, for NO_3_^−^ after the direct plasma treatment of distilled water.

### 2.3. Detection of Galantamine Standard by MALDI-IMS Using Two Different Matrices

**Figure 3:**
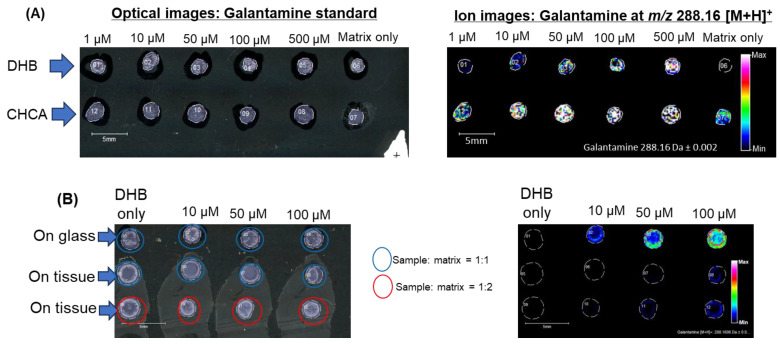
Detection of galantamine standards by MALDI-IMS. (**A**) Detection of galantamine standards on a glass slide using DHB or CHCA as the matrix. (**B**) Detection of galantamine standards spotted on a glass slide or in brain sections using DHB as the matrix.

To ensure the proper detection of galantamine by MALDI-IMS, this study used two parameters. (1) To check there was a suitable matrix for the MALDI-IMS detection of galantamine, two different matrices, a 2,5-dihydroxybenzoic acid (DHB) matrix and alpha-cyano-4-hydroxy-cinnamic acid (CHCA) matrix, were used to observe a galantamine standard spotted on a standard glass slide. Then, 0.3 µL/spot of standard galantamine was applied to the glass slide, followed by an additional 0.3 µL/spot of matrix solution. The ion image shows the detection of galantamine with both matrixes (Figure 3A). However, the ion image of the CHCA matrix alone showed a signal that overlaps with galantamine. This implies that the DHB matrix is more suitable for the MALDI detection of galantamine. (2) To check the MALDI-IMS detection of standard galantamine in brain tissues, the brain tissue was sectioned into 10 µm thicknesses and mounted on glass slides. The DHB matrix was used. Then, 0.3 µL/spot of standard galantamine was applied to the glass slide and the brain tissues, followed by an additional 0.3 µL/spot of the DHB matrix. Figure 3B shows that on the glass slide, galantamine was detected at all concentrations. However, the detection of galantamine in the brain section was noticeable at a 100 µM concentration and a sample–matrix ratio of 1:1, while for a sample–matrix ratio of 1:2, galantamine was detected at 50 µM and 100 µM concentrations (Figure 3B).

### 2.4. MALDI-IMS Detection of Galantamine in the Left and Right Hemispheres of the Brain

**Figure 4:**
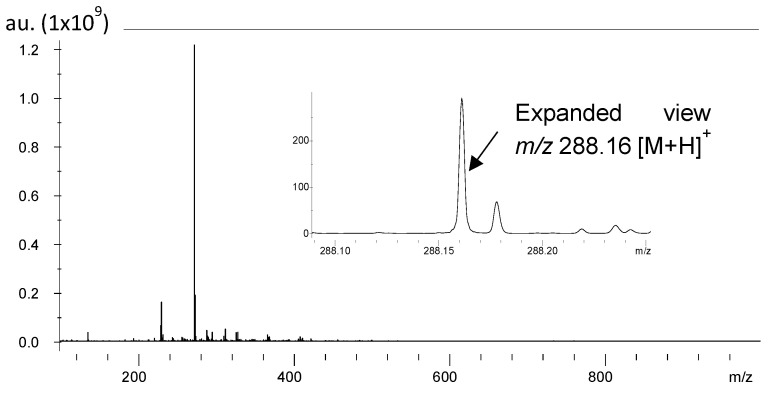
MALDI overall mass spectra for detecting galantamine in brain sections at 288.16 *m/z*.

**Figure 5:**
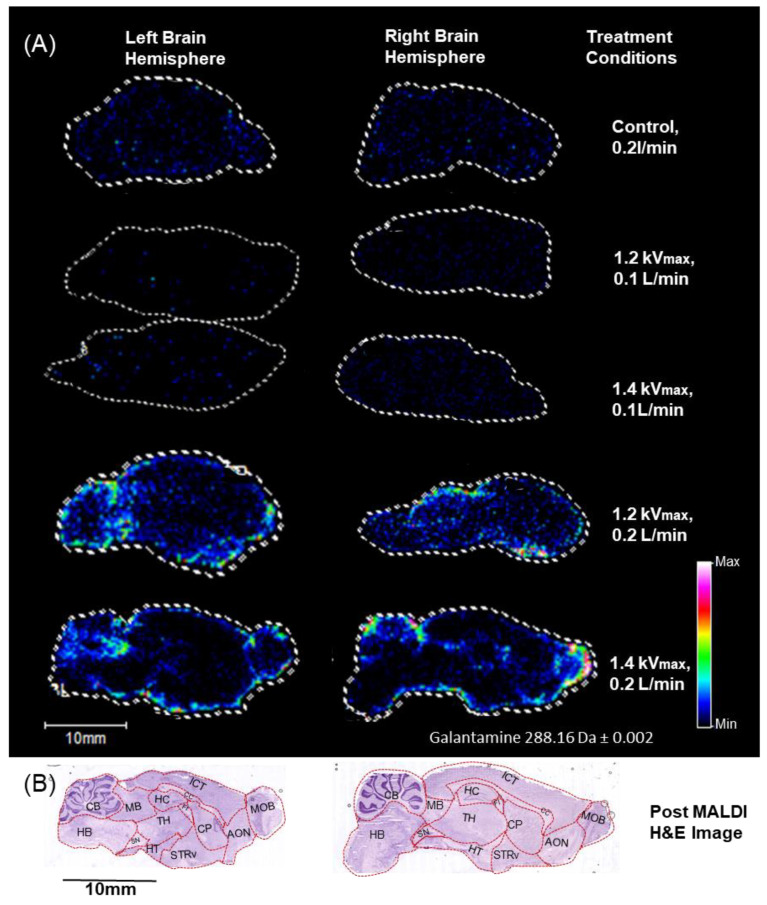
(**A**) MALDI-IMS ion image of galantamine distribution in the left and right hemispheres of the brain. (**B**) Hematoxylin and Eosin staining of brain sections. MOB: Main Olfactory Bulb; AON: Anterior Olfactory Nucleus; STRv: Stratum Ventral Region; CP: Caudate Putamen; CC: Corpus Callosum; ICT: Isocortex; HC: Hippocampus; FI: Fimbria of the hippocampus; TH: Thalamus; HT: Hypothalamus; SN: Substantia Nigra; MB: Midbrain; HB: Hindbrain; CB: Cerebellum.

**Figure 6:**
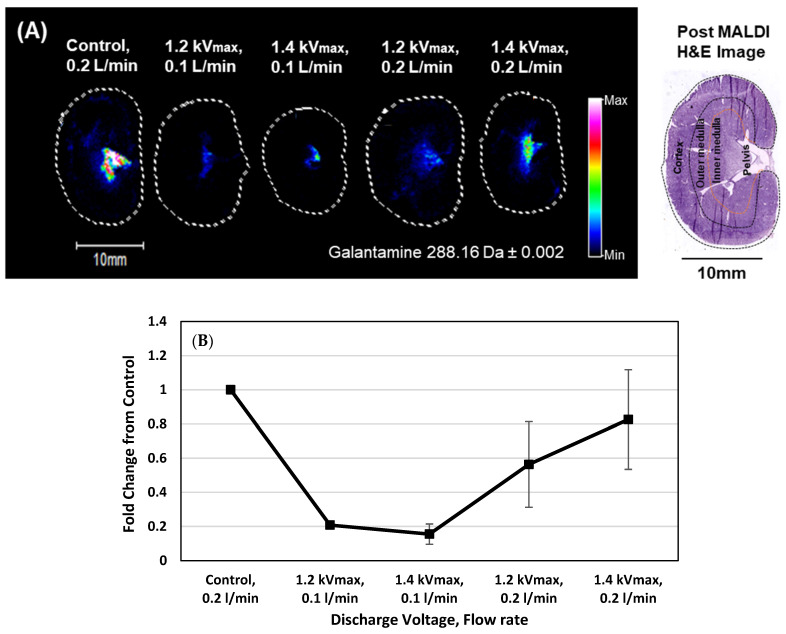
(**A**) Ion image of galantamine distribution in the kidney, detected by MALDI-IMS; (**B**) fold change measured as a ratio of plasma-treated kidney sections against non-plasma-treated (control) kidney sections.

Similarly to the standard, we acquired MALDI-IMS data using a solariX XR 7.0T from the brain section of the control and plasma-treated rats (Figure 4, Figure 5 and Figure 6A). Galantamine was also detected as protonated ions at *m/z* 288.16 [M+H]^+^ in the tissue sections. Figure 4 showed the mass spectra peaks of a brain section in the *m/z* range of 100–1000. The expanded view shows the galantamine detected at *m/z* 288.16. The mass spectrum of galantamine indicates the region in which galantamine was detected in the tissue sections.

Figure 5 shows the ion image of galantamine’s distribution in the left and right hemispheres of the brain, as detected by MALDI-IMS. The rat samples used in the experiment were treated for 4 min, followed by the immediate administration of galantamine under the following conditions: (1) control, with 0.2 L/min air flown through the nose. (2) 1.2 kV plasma, with 0.1 L/min air flown through the nose. (3) 1.4 kV plasma, with 0.1 L/min air flown through the nose. (4) 1.2 kV plasma, with 0.2 L/min air flown through the nose. (5) 1.4 kV plasma, with 0.2 L/min air flown through the nose. Galantamine was detected in all treated samples, but its most prominent distribution was observed in the plasma discharge at 1.4 kV and 0.2 L/min followed by 1.2 kV and 0.2 L/min. This suggests a dose-dependent effect of the plasma treatment. Furthermore, increasing the air flow rate from 0.1 L/min to 0.2 L/min resulted in an increase in the level of galantamine detected in the brain. In both the left and right hemisphere of the brain, galantamine was detected most strongly after the plasma treatment with a higher air flow rate of 0.2 L/min. The detected galantamine was most prominent in the olfactory bulb and the cerebellum area. These regions are clearly seen in the histological staining of the post-MALDI image using Hematoxylin and Eosin (H&E) and were visualized with NanoZoomer (Hamamatsu Photonics, Hamamatsu, Japan).

**Figure 7:**
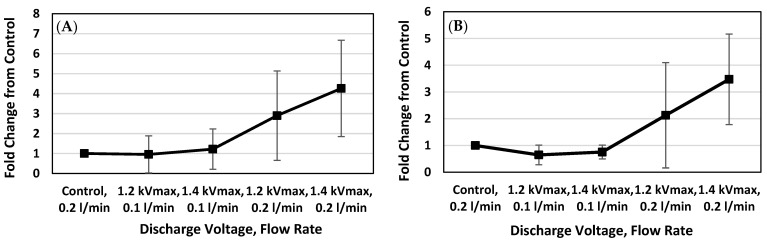
(**A**) The fold change in galantamine abundance in the left hemisphere of the brain, measured as a ratio of plasma-treated brain sections against non-plasma-treated (control) brain sections. (**B**) The fold change in galantamine abundance in the right hemisphere of the brain, measured as a ratio of plasma-treated sections against control sections.

In Figure 6B and Figure 7A,B, the fold change was calculated from the maximum height of the spectra intensities. These spectra are shown in Figure 4. The height of the peak indicates the relative abundance of the different components of the tissue section. It is estimated by establishing the ratio of the values of the plasma-treated samples against the control sample. The result (Figure 7A,B) showed an increasing distribution of galantamine with an increase in the plasma treatment. As the gas flow increased to 0.2 L/min, the plasma treatment at 1.4 kV led to a higher galantamine distribution. The increasing pattern of galantamine was similar in both the left and right hemispheres of the brain.

### 2.5. MALDI-IMS Detection of Galantamine in Kidney

The kidneys were also collected and analyzed by MALDI to assess whether galantamine entered systemic circulation after its initial absorption in the brain. Galantamine was detected in all kidney sections (Figure 6A). The drug was more prominent in the pelvis region compared to the medulla and cortex regions. The largest detected distribution of the drug was observed in the control, followed by plasma treatments of 1.4 kV and 1.2 kV at 0.2 L/min. Its high distribution in the control indicates that there was more circulation of the drug in the blood. The fold change in Figure 6B also confirmed a higher value in the control compared to the plasma treatment.

## 3. Discussion

RONS generated by NTP have been well documented to play a crucial role in biomedical applications. Within the RONS generated, NO plays multiple roles in the central nervous system. NO has been reported to expand blood vessels and increase blood flow [53]; it protects neuronal cells from damage and serves as a messenger in activating intracellular pathways [54]. Based on this, NO_2_- and NO_3_-, the most stable oxidative products of NO, were observed to be generated by our spiral DBD microplasma treatment. Their concentration increases with the increase in voltage and air flow. This may contribute to the therapeutic effect observed in plasma-treated rat samples. This is an indication that spiral DBD microplasma inhalation therapy could be a complementary approach to increasing the dose concentration of treatments for brain diseases in the brain. Spiral DBD microplasma is also a novel contribution to existing DBD plasma designs.

In our previous study of transdermal drug delivery [55], we identified the presence and effect of many highly reactive species (ROS, RNS, etc.) generated by microplasma on Yucatan micropig skin. Distortion of the stratum corneum lipid was observed. The study confirmed that plasma discharge improved the skin permeation and transdermal absorption of drugs without causing undesirable damage to the skin [55]. It was expected to minimize physical pain. Many in vitro studies have reported the neuroprotective effect of several plasma treatments [56]. However, how to use plasma to enhance in vivo brain drug delivery and translate this to real-world clinical disease treatment methods remains unknown.

In this study, we observed the N2B delivery of galantamine in male SD rats with the assistance of a spiral DBD microplasma. Male rats were used in this study to maintain hormonal consistency and avoid sex-based differences and weight differences. Our microplasma device was designed and optimized for the inhalation of the plasma components released during treatment. The plasma electrode was inserted to a depth of 2 mm and energized for 4 min. Immediately after that, galantamine was administered to both nostrils using a micropipette technique. The results showed that the inhalation of the spiral DBD microplasma discharge enhanced the delivery of galantamine to the brain. Also, increasing the air flow rate contributed to a larger amount of plasma species being inhaled. As expected, plasma is generated by the ionization of gas. Therefore, the more gas there is, the more plasma is generated. In both left- and right-brain sections, galantamine was most detected in plasma-treated rats subjected to the higher air flow rate of 0.2 L/min. There are four main plasma components that are released during treatment. They interact with other molecules in cells to create notable changes. These components include charged particles (ions and electrons), electromagnetic fields, photons (including UV light), and reactive species (RONS). These plasma component interactions in the nasal cavity increased epithelial permeability to allow deeper diffusion of the drug into the olfactory epithelium. This resulted in a better distribution of galantamine in the brain, though it was mostly concentrated at the olfactory bulb and the cerebellum. Based on the anatomy of rat noses, an electrode depth of 2 mm in the nose corresponded to drug delivery through the respiratory region; however, the plasma discharge effect indirectly influenced the adjacent olfactory region to enhance the permeation of galantamine. The localization of galantamine in the olfactory bulb of plasma-treated rats (Figure 5A) suggests that the drug entered the brain through the olfactory route. This is because the olfactory bulb is closely connected to the olfactory epithelium, which is responsible for the direct transport of drugs to the brain through the olfactory nerve pathway [57]. It is most likely that the plasma discharge enhanced local epithelial permeability, enabling the transport of galantamine from the nasal cavity to the brain through the olfactory nerve. Additionally, in the ion image in Figure 5A, the distribution of galantamine in the brain sections of the control rat with an air flow rate of 0.2 L/min was greater than that of plasma-treated rat with an air flow rate of 0.1 L/min. These results suggest that the air flow rate has a potential impact in distorting the nasal epithelia to allow the delivery of galantamine. In the kidney (Figure 6A,B), galantamine was highly detected in the kidney section of the control without plasma treatment, suggesting systemic absorption through the gastrointestinal tract (GI). This is an indication that the intranasally administered drug that was partially swallowed through the GI migrated to the blood and subsequently accumulated in the kidney. In contrast, in the plasma-treated group, there was a reduced level of galantamine in the kidney. This suggests that plasma discharge enhances drug delivery to the brain, reducing the systemic absorption of the drug through the GI.

## 4. Materials and Methods

### 4.1. Spiral DBD Plasma Electrode

**Figure 8:**
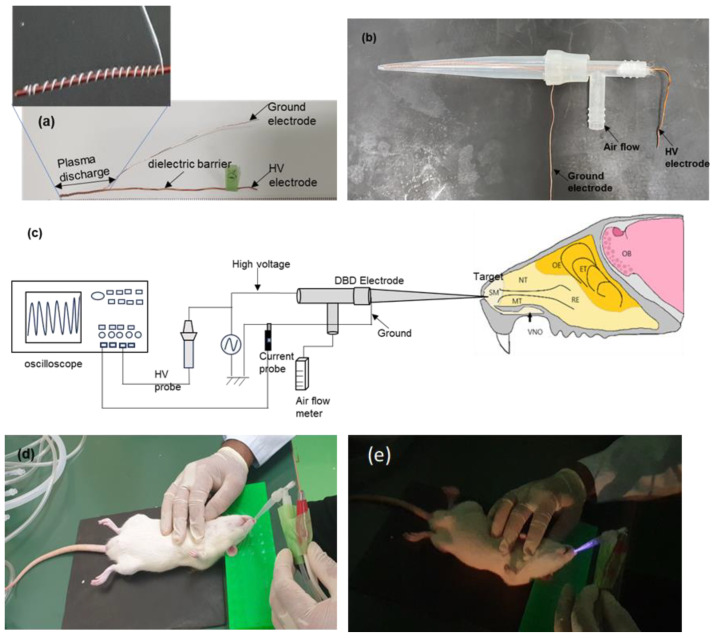
The spiral DBD plasma device used for enhancing the nose-to-brain distribution of galantamine in rats. (**a**) Electrode structure, (**b**) spiral DBD plasma device, (**c**) structure of the experimental setup, (**d**) position of rat under anesthesia with electrode in the left nostril, and (**e**) direct plasma treatment for intranasal delivery of galantamine with the electrode energized in air, showing the radiative state of the plasma discharge observed in a dark room.

The spiral DBD microplasma electrode used for assisting in the N2B delivery of galantamine to SD rats is shown in Figure 8a. The DBD microplasma electrode was made with a nanocomposite vanish wire with a thickness of 0.5 mm and a length of 10 cm. The thickness of the conducting wire and dielectric coating are 0.481 mm and 22.9 μm, respectively. A copper wire with a thickness of 0.2 mm was wound around the nanocomposite wire with a winding gap of 2 mm. The total winding length was 3 cm, and this is the plasma discharge length. The spiral DBD plasma electrode tip was covered with silicon glue to prevent current jumping. It was further placed inside a polypropylene pipette tip with an opening for gas flow, as shown in Figure 8b. The distance between the spiral electrode’s tip and the pipette tip was 0.3 mm. The working gas employed was 100% room air. The dielectric barrier coating of the electrode covering the region of the high-voltage (HV) electrode was peeled off. The electrode was driven by a sinusoidal neon power transformer (αNEON M-1H) with frequency of 27 kHz. The wound copper wire served as the ground (GND) electrode. The spiral DBD plasma electrode was used at atmospheric pressure in room air. The room air flow rate was controlled with a flow meter, ranging from 0.1 L/min to 1 L/min.

### 4.2. Plasma Characteristics and Treatment Setup

The electrical waveform was measured and recorded with an oscilloscope (Tektronix, TDS 2014B, Beaverton, OR, USA). The oscilloscope was linked to a high-voltage probe (Tektronix, P6105A) and Pearson current monitor to measure the discharge voltage and discharge current of the spiral DBD microplasma electrode when energized. Figure 8c shows the experimental setup. The electrode surface temperature was measured with a thermal video system (Avio handy thermos TVS-200, Nippon Avionics Co., Ltd., Kanagawa, Japan).

The optimum experimental conditions were determined by adjusting the gas flow rate and the applied voltage. A model rat was treated with the spiral DBD plasma electrode to optimize its conditions for enhancing the N2B distribution of drug. The rat was laid on its back on a flat surface and its head kept at an angle of 45 °C on a head support (Figure 8d). Figure 8e shows the spiral electrode, which was inserted into the rat’s nose to a depth of 2 mm and energized. Figure 8e shows the energized state of the plasma treatment, with its brights streams of atoms and molecules in a radiative state. This was followed by the immediate administration of galantamine across the left and right nostrils using a micropipette method.

### 4.3. Animals

This study was carried out at Hamamatsu University School of Medicine and in compliance with article 11 paragraph 1 of the regulation on animal experiments. A total of 27 male Sprague Dawley (SD) rats (8 weeks), with weights between 250 and 300 g, were purchased from Japan SLC Inc. (Hamamatsu, Japan). The rats were kept in a temperature-controlled room with a 12 h light–dark cycle. The rats were allowed to eat and drink freely. This study ensured that animal suffering was minimized.

### 4.4. Galantamine Concentration and Administration

#### 4.4.1. Preparation of Galantamine Solution

Galantamine HBr is a white powder that is soluble in water [58]. It has a molecular weight of 368.27 Da. To make a concentration of 100 mM, 36 mg of galantamine hydrobromide was completely dissolved in 1 mL of warm distilled water. The solution was vortexed until a clear solution was obtained.

#### 4.4.2. Microplasma Treatment Conditions for N2B Galantamine HBr Administration

Room air was used as the working gas. The plasma treatment time was 4 min in the rats’ left nostril only, followed by the immediate administration of galantamine. A total of 10 µL of galantamine HBr was added to each nostril in drops of 2 µL, alternating between the left and right nostril. The concentration of drug administered was 0.368 mg/nose. This concentration is within the doses reported by Moschonas et al. for evaluating the efficacy chronic galantamine in a pre-clinical rat model of traumatic brain injury [59]. This experiment was organized into 5 conditions and 4 sets (n = 4). The conditions were as follows:

- Control: 0.2 L/min flow rate with no plasma discharge;
- 1.2 kVmax plasma discharge at 0.1 L/min air flow rate;
- 1.2 kVmax plasma discharge at 0.2 L/min air flow rate;
- 1.4 kVmax plasma discharge at 0.1 L/min air flow rate;
- 1.4 kVmax plasma discharge at 0.2 L/min air flow rate.

The experiment was conducted in 4 sets of trials that were completed at different times. In each set of the experiments, 1 rat was used per group. This means that 20 rats were used for the 4 experimental sets, which each contained 5 groups. Seven rats were excluded from this experiment. This includes rats whose brains were damaged during collection, those tested for intraperitoneal injections, and those tested for the application of an atmospheric plasma jet with no plume generated.

#### 4.4.3. Plasma Treatment and Intranasal Administration of Galantamine HBr

Prior to treatment, the rats’ weight was measured. The rats were sedated in an isoflurane chamber with 3% isoflurane at 1 L/min, followed by an intraperitoneal injection (I.P) of the three mixes of anesthesia shown in Table 1. The volume of anesthesia injected in each rat was based on their individual measured weight (Table 2). The anesthetized rats were kept in a supine position with their head inclined on a support. The plasma device was inserted into the left nostril at a depth of approximately 2 mm and energized for 4 min. This was followed by the immediate administration of galantamine HBr, using the micropipette method [10], in drops of 2 µL alternating between the left and right nostril until a total volume of 10 µL (0.368 mg) per nostril was recorded. The administration of the drug in small drops was to prevent nasal irritation, which could lead to reduced drug uptake [57,60]. The rats remained in the same supine position for 10 min before returning to their cage. The rats stayed in the cage for an additional hour before euthanasia with 5% isoflurane at 1 L/min. Once the rat exhibited a complete lack of reflexes, its head was taken off using a surgical instrument. The rats’ brain and kidney were collected following laboratory procedures [61] and frozen with dry ice. They were then stored at −80 °C until cryosection and data acquisitions by MALDI-IMS.

### 4.5. Preparation of Galantamine Standard Solution for MALDI-IMS Measurement

The MALDI source and data acquisition parameters were optimized, and the MALDI-IMS data were acquired in positive ion mode using a solariX XR 7.0 T. The *m/z* range was set between 100 and 1000. Prior to the animal experiments, MALDI-IMS conditions were optimized for the detection of standard galantamine. The standard galantamine was prepared as a stock solution of 50 mM in ethanol–water (50:50, *v*/*v*). It was further diluted with methanol–water (50:50, *v*/*v*) to obtain 500 µM, 100 µM, 50 µM, 10 µM, and 1 µM concentrations.

### 4.6. Tissue Sectioning and MALDI-IMS Data Acquisition

A sagittal section method was adopted. Sections with a thickness of 10 µm were prepared from the brain and kidney using a CM1950 cryostat (Leica Biosystems, Wetzlar, Germany) at a cutting temperature of −20 °C and mounted on a pre-cooled 100 Ω Indium Tin Oxide (ITO)-coated slide (Matsunami, Osaka, Japan). Afterwards, the ITO-coated slide was dried at room temperature using a dry vacuum pump (ULVAC DTC-21). Furthermore, matrix was deposited on the surface of the samples using a TM-Sprayer (HTX Technologies, Chapel Hill, NC, USA) connected to a syringe pump. The spraying conditions were as follows: 70 °C nozzle temperature, 24 (CC pattern) passes, 50 µL/min matrix flow rate, pressure of 10 psi, and nozzle height of 40 mm. After matrix spraying, the samples were subjected to data acquisition using a 7.0T SolariX XR equipped with a superconducting magnet (7.0 T) and a Smartbeam II™ laser (355 nm) unit (Bruker Daltonics, Bremen, Germany). One tissue section was prepared per experimental condition. The MALDI-IMS data were acquired in positive ionization mode. The following parameters were set for data acquisition: the mass range was 100–1000 *m/z*, raster width was 250 µm, laser power efficiency was 45%, laser focus was large, laser shot was 100, frequency was 1000 Hz, data magnitude size was 1 M, and ToF was 0.7 ms.

### 4.7. Hematoxylin and Eosin (H&E) Staining and Scanning

Hematoxylin and Eosin (H&E) staining of the post-MALDI-IMS tissue sections was performed to visualize the microanatomy of the tissues. The H&E staining of the tissue sections mounted on the ITO-coated slides started with pre-washing the slide with acetone for a few seconds to remove the DHB matrix. The slide was dipped in Hematoxylin for 5 min, and then washed with tap water for 3 min. The slide was further dipped in 80% ethanol for 1 min, then immersed in Eosin for 30 s to create color, and washed again with water for 1 min. The tissue sections were then dehydrated by passing them through 80%, 90%, 100%, and another 100% concentration of ethanol for 1 min per concentration. The slide was removed and immersed in xylene for 3 min to remove any water content left in the tissue sections. Finally, pathomount (a mounting medium) was added to protect the tissues from oxidation. The samples were then attached to a glass slide of a 0.12–0.17 mm thickness.

Digital scanning and imaging was performed with a Nanozoomer S60 (Hamamatsu Photonics, Hamamatsu, Japan).

## 5. Conclusions

The application of dielectric barrier discharge plasma to enhance the nose-to-brain delivery of galantamine in a rat model was established as a preliminary evaluation of this delivery mechanism for the treatment of neurological diseases. We designed a spiral DBD plasma that is easily used in a rat’s nose. The applied voltage, gas flow, and treatment conditions were optimized for the treatment of rats. All treated rats were alive until euthanasia. Inhalation of the plasma components released during the plasma treatment increased the N2B delivery of galantamine to the rat brain. Though the plasma treatment was performed in the left nostril of the rat and galantamine was administered in both the left and right nostrils, we evaluated the distribution of galantamine in both the left and right hemispheres of the brain using MALDI-IMS. Galantamine was detected in both brain hemispheres. These results indicate an enhancement in drug delivery to both brain hemispheres regardless of the nostril treated with the plasma discharge. The spiral DBD microplasma technique offers a promising and innovative approach to N2B drug delivery to the brain. However, compared to other advanced techniques like nanoparticles and focused ultrasound (FUS), which are designed to cross the blood–brain barrier, the mechanism of action of this method is the generation of reactive plasma species that temporarily increase the nasal epithelial permeation of drugs to the brain. Additionally, in terms of technological simplicity, this microplasma technique has a simple design that involves an electrode capable of plasma species generation, whereas nanoparticles and FUS require advanced formulation technology and imaging guidance.

Healthy model SD rats were used in this study to avoid the enzymatic activities present in disease models, which may affect the distribution of galantamine in the brain. Thus, more preclinical studies are required that use disease-specific rat models to validate the clinical applications of these findings. These future studies will address the translational gap. Our findings suggest that the inhalation of the plasma components released during this treatment has the potential to enhance the delivery of galantamine to the brain for the treatment of AD.

## Figures and Tables

**Table 1:** Triple anesthesia mixture and the concentrations used for a 5 mL solution.

	Medetomidine	Midazolam	Butorphanol	Saline
Dosage (0.05 mL/g)	0.3 mg/kg	4 mg/kg	5 mg/kg	
Preparing 5 mL	0.3 mL	0.8 mL	1 mL	2.9 mL

**Table 2:** The triple anesthesia dosing volume, based on the weight of the rats.

Rat weight (g)	200	220	240	260	280	300	320
Anesthesia dose (mL)	0.50	0.55	0.60	0.65	0.70	0.75	0.80

## Data Availability

Data are contained within the article.
